# Disparities in COVID-19 Vaccination Coverage Between Urban and Rural Counties — United States, December 14, 2020–April 10, 2021

**DOI:** 10.15585/mmwr.mm7020e3

**Published:** 2021-05-21

**Authors:** Bhavini Patel Murthy, Natalie Sterrett, Daniel Weller, Elizabeth Zell, Laura Reynolds, Robin L. Toblin, Neil Murthy, Jennifer Kriss, Charles Rose, Betsy Cadwell, Alice Wang, Matthew D. Ritchey, Lynn Gibbs-Scharf, Judith R. Qualters, Lauren Shaw, Kathryn A. Brookmeyer, Heather Clayton, Paul Eke, Laura Adams, Julie Zajac, Anita Patel, Kimberley Fox, Charnetta Williams, Shannon Stokley, Stephen Flores, Kamil E. Barbour, LaTreace Q. Harris

**Affiliations:** ^1^Immunization Services Division, National Center for Immunization and Respiratory Diseases, CDC; ^2^CDC COVID-19 Response Team; ^3^Stat-Epi Associates, Inc., Ponte Vedra Beach, Florida.

Approximately 60 million persons in the United States live in rural counties, representing almost one fifth (19.3%) of the population.* In September 2020, COVID-19 incidence (cases per 100,000 population) in rural counties surpassed that in urban counties (*1*). Rural communities often have a higher proportion of residents who lack health insurance, live with comorbidities or disabilities, are aged ≥65 years, and have limited access to health care facilities with intensive care capabilities, which places these residents at increased risk for COVID-19–associated morbidity and mortality (*2*,*3*). To better understand COVID-19 vaccination disparities across the urban-rural continuum, CDC analyzed county-level vaccine administration data among adults aged ≥18 years who received their first dose of either the Pfizer-BioNTech or Moderna COVID-19 vaccine, or a single dose of the Janssen COVID-19 vaccine (Johnson & Johnson) during December 14, 2020–April 10, 2021 in 50 U.S. jurisdictions (49 states and the District of Columbia [DC]). Adult COVID-19 vaccination coverage was lower in rural counties (38.9%) than in urban counties (45.7%) overall and among adults aged 18–64 years (29.1% rural, 37.7% urban), those aged ≥65 years (67.6% rural, 76.1% urban), women (41.7% rural, 48.4% urban), and men (35.3% rural, 41.9% urban). Vaccination coverage varied among jurisdictions: 36 jurisdictions had higher coverage in urban counties, five had higher coverage in rural counties, and five had similar coverage (i.e., within 1%) in urban and rural counties; in four jurisdictions with no rural counties, the urban-rural comparison could not be assessed. A larger proportion of persons in the most rural counties (14.6%) traveled for vaccination to nonadjacent counties (i.e., farther from their county of residence) compared with persons in the most urban counties (10.3%). As availability of COVID-19 vaccines expands, public health practitioners should continue collaborating with health care providers, pharmacies, employers, faith leaders, and other community partners to identify and address barriers to COVID-19 vaccination in rural areas (*2*).

Data on COVID-19 vaccine doses administered in the United States are reported to CDC by jurisdictions, pharmacies, and federal entities through immunization information systems (IISs),^†^ the Vaccine Administration Management System,^§^ or direct data submission.^¶^ Adults aged ≥18 years with a valid county of residence in one of 49 states or DC who received their first COVID-19 vaccine dose** during December 14, 2020–April 10, 2021, and whose data were reported to CDC by April 15, 2021, were included in the analysis.^††^ COVID-19 vaccine doses administered to persons living in Hawaii and in eight counties in California with <20,000 residents were excluded, because these states have data-sharing restrictions on county-level information reported to CDC. Vaccine doses administered to persons living in U.S. territories were also excluded because territorial jurisdictional divisions could not be mapped to urban-rural classifications at the county level.

First doses of COVID-19 vaccine were matched by county of residence to one of six urban-rural categories according to the 2013 National Center for Health Statistics (NCHS) urban-rural classification scheme. To further classify counties into two categories (urban versus rural), four of these six categories (large central metropolitan, large fringe metropolitan, medium metropolitan, and small metropolitan) were combined into urban areas, and two (micropolitan and noncore) were combined into rural areas (*4*).

Vaccination coverage for adults aged ≥18 years was calculated overall and by age group (18–64 and ≥65 years), sex, jurisdiction, and two- and six-level urban-rural classification. Coverage by race and ethnicity was not calculated because information on race and ethnicity was missing for 40% of data. Population size was obtained by county, age group, and sex from the U.S. Census Bureau’s 2019 Population Estimates Program (*5*). Because only the first dose of a 2-dose vaccination series or the only dose for a single-dose vaccine were analyzed, the total number of doses allowed per county was capped at the population size of the county.^§§^ The percentage of persons who traveled outside their county of residence for vaccination was calculated at the national level and stratified by jurisdiction for both the two- and six-level urban-rural classifications. Tests for statistical significance were not conducted because the data represent the U.S. population (minus Hawaii and eight counties in California) and were not based on population samples.

First-dose COVID-19 vaccination coverage was lower in rural than in urban counties for adults overall (38.9% rural, 45.7% urban) (Table); for adults aged 18–64 years (29.1% rural, 37.7% urban) and for those aged ≥65 years (67.6% rural, 76.1% urban); for women (41.7% rural, 48.4% urban); and for men (35.3% rural, 41.9% urban). Among jurisdictions, coverage varied by urban-rural classification; in 36 (72%) jurisdictions, coverage was higher in urban counties, in five (10%) coverage was higher in rural counties, and in five (10%) coverage was similar (i.e., within 1%) in both urban and rural counties. Vaccination coverage by urban-rural classification could not be calculated for four jurisdictions that had no rural counties.

**TABLE:** Vaccination coverage among adults aged ≥18 years who received their first dose of COVID-19 vaccine,* by jurisdiction, sex, age group, and urban-rural classification^†^ — United States,^§^ December 14, 2020–April 10, 2021

Jurisdiction	No. (%) vaccinated								
	Overall	Six-level urban-rural classification						Two-level urban-rural classification	
		Large central metropolitan	Large fringe metropolitan^¶^	Medium metropolitan	Small metropolitan	Micropolitan	Noncore	Urban	Rural
**United States**	**113,554,259 (44.7)**	**37,075,718 (47.1)**	**29,206,614 (45.8)**	**23,861,372 (45.4)**	**9,505,176 (40.9)**	**8,368,195 (39.7)**	**5,537,184 (37.8)**	**99,648,880 (45.7)**	**13,905,379 (38.9)**
Alabama	1,294,410 (33.9)	221,812 (43.6)	99,898 (26.1)	366,648 (36.1)	336,235 (33.0)	124,904 (30.5)	144,913 (30.4)	1,024,593 (35.0)	269,817 (30.4)
Alaska	273,888 (49.7)	—**	—**	148,209 (49.6)	31,389 (42.5)	22,787 (63.1)	71,503 (50.0)	179,598 (48.2)	94,290 (52.6)
Arizona	2,514,666 (44.6)	1,444,473 (42.1)	137,650 (38.2)	442,370 (53.2)	333,991 (44.6)	109,132 (58.1)	47,050 (61.1)	2,358,484 (43.9)	156,182 (59.0)
Arkansas	838,457 (36.2)	—**	10,405 (29.7)	432,025 (38.7)	96,973 (33.2)	149,145 (34.1)	149,909 (34.3)	539,403 (37.4)	299,054 (34.2)
California	15,349,193 (50.3)	10,168,806 (51.7)	1,986,161 (49.5)	2,543,570 (47.4)	385,248 (42.3)	200,457 (44.1)	64,951 (45.4)	15,083,785 (50.4)	265,408 (44.4)
Colorado	2,177,824 (48.4)	301,043 (51.1)	838,482 (48.6)	649,740 (47.7)	106,506 (42.2)	164,065 (51.3)	117,988 (47.0)	1,895,771 (48.3)	282,053 (49.4)
Connecticut	1,565,628 (55.2)	390,071 (55.3)	147,437 (56.9)	945,420 (54.8)	—**	82,700 (55.9)	—**	1,482,928 (55.1)	82,700 (55.9)
Delaware	376,448 (48.9)	—**	215,689 (49.1)	106,497 (55.7)	54,262 (38.9)	—**	—**	376,448 (48.9)	—**
District of Columbia	272,747 (47.2)	272,747 (47.2)	—**	—**	—**	—**	—**	272,747 (47.2)	—**
Florida	7,558,301 (43.8)	2,612,865 (43.8)	2,098,598 (43.9)	2,181,338 (44.4)	487,184 (48.1)	98,684 (33.7)	79,632 (28.2)	7,379,985 (44.3)	178,316 (31.0)
Georgia	1,570,189 (19.4)	188,126 (22.5)	725,542 (19.4)	248,864 (26.8)	226,829 (18.7)	115,309 (15.0)	65,519 (10.3)	1,389,361 (20.7)	180,828 (12.9)
Idaho	545,857 (40.8)	—**	—**	250,086 (44.3)	135,313 (39.9)	120,446 (37.3)	40,012 (35.9)	385,399 (42.6)	160,458 (36.9)
Illinois	4,798,337 (48.7)	2,024,718 (50.2)	1,573,694 (50.0)	339,073 (48.5)	381,873 (46.1)	291,296 (43.1)	187,683 (40.4)	4,319,358 (49.6)	478,979 (42.0)
Indiana	2,057,161 (39.8)	263,355 (36.2)	727,655 (43.9)	319,160 (42.6)	342,196 (37.5)	281,902 (36.6)	122,893 (35.2)	1,652,366 (40.9)	404,795 (36.1)
Iowa	1,187,572 (48.9)	—**	—**	461,103 (49.4)	267,887 (51.4)	174,447 (46.2)	284,135 (47.6)	728,990 (50.1)	458,582 (47.1)
Kansas	1,041,465 (47.1)	—**	367,647 (54.6)	208,267 (43.0)	179,347 (49.3)	165,309 (41.2)	120,895 (41.5)	755,261 (49.7)	286,204 (41.3)
Kentucky	1,523,875 (44.0)	313,875 (52.5)	230,936 (43.6)	286,818 (50.6)	136,493 (39.4)	266,331 (39.9)	289,422 (38.2)	968,122 (47.4)	555,753 (39.0)
Louisiana	1,343,593 (37.7)	165,679 (53.0)	308,584 (45.6)	484,513 (36.2)	217,680 (32.7)	88,773 (29.9)	78,364 (29.0)	1,176,456 (39.3)	167,137 (29.5)
Maine	575,911 (52.6)	—**	—**	244,914 (55.7)	101,952 (48.6)	49,521 (50.0)	179,524 (51.7)	346,866 (53.4)	229,045 (51.3)
Maryland	2,300,883 (48.8)	191,933 (40.5)	1,875,332 (50.6)	111,818 (42.4)	62,256 (43.0)	29,755 (53.5)	29,789 (45.4)	2,241,339 (48.8)	59,544 (49.1)
Massachusetts	2,611,958 (47.1)	306,989 (45.7)	1,707,107 (51.1)	518,678 (44.6)	49,567 (17.4)	29,535 (40.8)	82 (0.9)	2,582,341 (47.3)	29,617 (36.4)
Michigan	3,414,578 (43.5)	768,690 (41.8)	1,051,335 (44.6)	579,511 (44.3)	385,356 (42.9)	391,849 (43.4)	237,837 (44.2)	2,784,892 (43.5)	629,686 (43.7)
Minnesota	2,121,068 (48.9)	736,642 (52.2)	562,068 (43.8)	108,924 (57.5)	241,769 (49.4)	258,667 (50.1)	212,998 (47.7)	1,649,403 (48.9)	471,665 (49.0)
Mississippi	828,073 (36.4)	—**	65,816 (32.7)	298,780 (39.9)	39,999 (34.9)	257,322 (36.0)	166,156 (33.4)	404,595 (38.0)	423,478 (34.9)
Missouri	1,843,060 (38.7)	320,345 (40.9)	800,596 (43.5)	137,447 (35.9)	213,023 (38.0)	174,846 (31.3)	196,803 (30.6)	1,471,411 (41.3)	371,649 (31.0)
Montana	372,927 (44.4)	—**	—**	—**	139,699 (47.5)	109,748 (41.4)	123,480 (43.9)	139,699 (47.5)	233,228 (42.7)
Nebraska	718,993 (49.3)	—**	—**	450,638 (51.6)	40,185 (49.7)	112,525 (45.6)	115,645 (45.1)	490,823 (51.4)	228,170 (45.3)
Nevada	1,015,950 (42.6)	739,037 (42.3)	—**	179,391 (47.9)	21,360 (47.9)	67,238 (34.2)	8,924 (34.5)	939,788 (43.4)	76,162 (34.2)
New Hampshire	605,093 (54.8)	—**	204,277 (57.1)	158,455 (47.6)	—**	214,430 (57.5)	27,931 (67.4)	362,732 (52.5)	242,361 (58.5)
New Jersey	3,516,994 (50.7)	728,029 (46.3)	2,408,516 (52.4)	287,894 (49.4)	92,555 (48.7)	—**	—**	3,516,994 (50.7)	—**
New Mexico	943,664 (58.2)	—**	—**	411,876 (57.3)	247,917 (65.1)	245,120 (54.6)	38,751 (53.0)	659,793 (60.0)	283,871 (54.4)
New York	7,449,653 (48.3)	3,646,082 (45.9)	2,201,835 (51.7)	759,849 (52.3)	342,276 (50.5)	366,736 (46.9)	132,875 (42.5)	6,950,042 (48.5)	499,611 (45.7)
North Carolina	3,497,654 (42.7)	807,462 (47.5)	384,965 (35.9)	1,326,943 (45.4)	302,213 (41.4)	485,062 (38.7)	191,009 (37.9)	2,821,583 (43.9)	676,071 (38.5)
North Dakota	266,915 (45.9)	—**	—**	—**	148,378 (49.9)	51,760 (37.9)	66,777 (45.1)	148,378 (49.9)	118,537 (41.7)
Ohio	3,980,433 (43.7)	1,225,497 (46.7)	876,242 (45.7)	1,053,437 (44.6)	146,003 (37.9)	558,642 (37.7)	120,612 (35.3)	3,301,179 (45.3)	679,254 (37.2)
Oklahoma	1,311,507 (43.6)	316,961 (53.3)	195,507 (41.6)	361,732 (43.9)	40,249 (41.6)	240,550 (39.6)	156,508 (38.1)	914,449 (46.0)	397,058 (39.0)
Oregon	1,494,454 (44.6)	332,259 (50.1)	393,659 (42.7)	286,399 (44.6)	247,465 (42.4)	194,569 (42.4)	40,103 (48.9)	1,259,782 (44.8)	234,672 (43.4)
Pennsylvania	4,817,265 (47.4)	1,098,792 (49.2)	1,556,236 (52.9)	1,328,061 (46.2)	382,538 (40.3)	327,898 (38.9)	123,740 (38.0)	4,365,627 (48.5)	451,638 (38.7)
Rhode Island	407,784 (47.7)	229,134 (45.1)	178,650 (51.5)	—**	—**	—**	—**	407,784 (47.7)	—**
South Carolina	1,575,298 (39.0)	—**	105,409 (33.4)	1,071,517 (39.5)	182,137 (43.3)	132,651 (37.6)	83,584 (35.7)	1,359,063 (39.4)	216,235 (36.8)
South Dakota	247,945 (37.1)	—**	—**	—**	128,452 (39.1)	64,444 (35.7)	55,049 (34.7)	128,452 (39.1)	119,493 (35.2)
Tennessee	2,032,692 (38.2)	533,687 (42.5)	383,619 (36.9)	559,029 (41.0)	168,327 (36.7)	223,523 (32.6)	164,507 (31.9)	1,644,662 (39.9)	388,030 (32.3)
Texas	9,325,215 (43.2)	4,562,747 (44.5)	1,848,021 (43.2)	1,575,372 (47.3)	502,046 (36.1)	458,287 (36.9)	378,742 (34.2)	8,488,186 (44.1)	837,029 (35.6)
Utah	1,039,555 (45.7)	423,864 (49.8)	19,552 (39.9)	390,166 (42.8)	98,050 (44.2)	63,274 (47.2)	44,649 (41.9)	931,632 (45.8)	107,923 (44.8)
Vermont	269,382 (52.8)	—**	—**	—**	93,365 (51.9)	110,027 (55.4)	65,990 (50.1)	93,365 (51.9)	176,017 (53.3)
Virginia	3,162,645 (47.4)	463,230 (43.6)	1,799,645 (50.0)	244,719 (45.6)	302,185 (47.0)	86,015 (41.3)	266,851 (42.5)	2,809,779 (48.1)	352,866 (42.2)
Washington	2,745,505 (46.1)	918,867 (51.0)	736,446 (42.9)	478,192 (42.9)	342,357 (48.2)	201,142 (42.3)	68,501 (51.2)	2,475,862 (46.3)	269,643 (44.3)
West Virginia	325,762 (22.7)	—**	11,485 (25.8)	58,693 (22.8)	139,026 (23.8)	53,402 (22.7)	63,156 (20.2)	209,204 (23.6)	116,558 (21.3)
Wisconsin	2,267,575 (49.8)	357,901 (49.7)	371,918 (51.0)	465,236 (57.3)	528,622 (48.0)	275,088 (44.4)	268,810 (47.0)	1,723,677 (51.2)	543,898 (45.6)
Wyoming	178,257 (40.1)	—**	—**	—**	56,443 (41.1)	78,882 (42.7)	42,932 (34.9)	56,443 (41.1)	121,814 (39.6)
**Demographic characteristics**									
**Sex**									
Male	50,684,095 (41.0)	16,606,553 (43.6)	12,926,239 (41.8)	10,606,151 (41.5)	4,266,165 (37.4)	3,756,557 (35.9)	2,522,430 (34.4)	44,405,108 (41.9)	6,278,987 (35.3)
Female	61,803,696 (47.4)	20,118,007 (49.6)	16,023,200 (48.8)	13,032,454 (48.2)	5,149,771 (43.5)	4,519,824 (42.5)	2,960,440 (40.5)	54,323,432 (48.4)	7,480,264 (41.7)
**Age group, yrs**									
18–64	73,245,975 (36.6)	25,903,354 (40.3)	18,997,421 (37.6)	14,910,642 (36.4)	5,681,032 (31.8)	4,798,499 (30.0)	2,955,027 (27.7)	65,492,449 (37.7)	7,753,526 (29.1)
≥65	40,147,289 (74.7)	11,035,258 (76.4)	10,205,186 (76.9)	8,949,648 (77.3)	3,812,281 (70.8)	3,566,401 (69.8)	2,578,515 (64.7)	34,002,373 (76.1)	6,144,916 (67.6)

* First dose of COVID-19 vaccine is defined either as the first of 2 doses for the Pfizer-BioNTech or Moderna vaccines, or a single dose for the Janssen (Johnson & Johnson) vaccine.

^†^ First doses of COVID-19 vaccine were matched by county of residence to one of six urban-rural categories according to the 2013 National Center for Health Statistics urban-rural classification scheme (https://www.cdc.gov/nchs/data/series/sr_02/sr02_166.pdf). To further classify counties into two categories (urban versus rural), four of these six categories were combined into urban areas (large central metropolitan, large fringe metropolitan, medium metropolitan, and small metropolitan) and two were combined into rural areas (micropolitan and noncore).

**^§^** Excludes doses with state of residence reported as Hawaii, a territory, an island, or a county of residence in California with population <20,000. Completeness of county data varied by jurisdiction. Three states (Georgia, South Dakota, and West Virginia) had <80% completeness for county of residence data.

^¶^ Large fringe metropolitan refers to suburban areas.

** Jurisdiction does not have any counties at this level of urban-rural classification.

Overall, 67.1% of vaccinated persons were vaccinated in their county of residence and 98.3% in their state of residence. The proportion of persons who traveled outside their county of residence for vaccination varied by jurisdiction, based on the two-level urban-rural classification (Figure 1). Analysis using the six-level urban-rural classification found that a larger proportion of persons in large fringe metropolitan counties (i.e., suburban areas) and noncore counties (i.e., the most rural areas) traveled to nonadjacent counties (i.e., farther from their county of residence) for vaccination (13.9% and 14.6%, respectively) compared with persons in the most urban counties (10.3%) (Figure 2).

**Figure 1:**
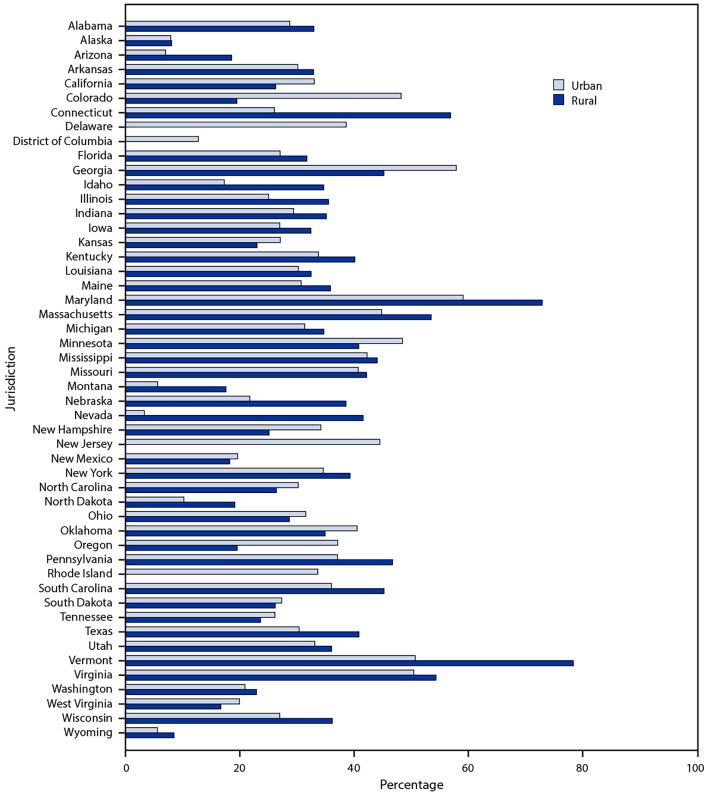
Percentage of vaccinated persons who traveled outside their county of residence* for their first dose of COVID-19 vaccine,^†^ by jurisdiction and urban-rural classification^§^ — United States, December 14, 2020–April 10, 2021 * Excludes doses with state of residence reported as Hawaii, a territory, an island, or a county of residence in California with population <20,000. Completeness of county data varied by jurisdiction. Three states (Georgia, South Dakota, and West Virginia) had <80% completeness for county of residence data. Four jurisdictions (Delaware, New Jersey, Rhode Island, and District of Columbia) did not have rural counties. ^†^ First dose of COVID-19 vaccine is defined either as the first of 2 doses for the Pfizer-BioNTech or Moderna vaccines, or a single dose for the Janssen (Johnson & Johnson) vaccine. **^§^** First doses of COVID-19 vaccine were matched by county of residence to one of six urban-rural categories according to the 2013 National Center for Health Statistics urban-rural classification scheme (https://www.cdc.gov/nchs/data/series/sr_02/sr02_166.pdf). To further classify counties into two categories (urban versus rural), four of these six categories were combined into urban areas (large central metropolitan, large fringe metropolitan, medium metropolitan, and small metropolitan) and two were combined into rural areas (micropolitan and noncore).

**Figure 2:**
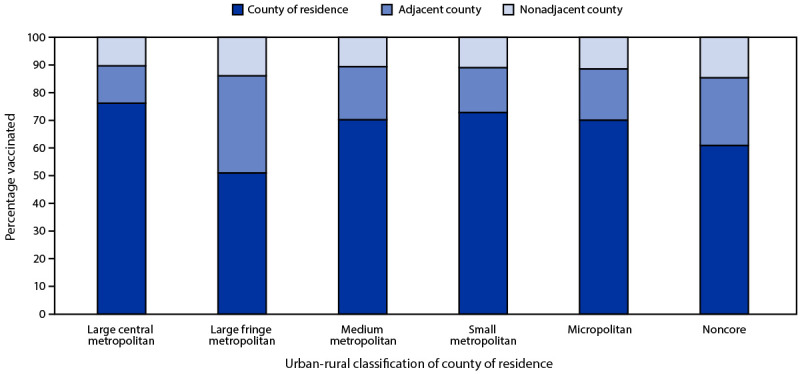
Location of receipt of first COVID-19 vaccine dose* among vaccinated persons, by urban-rural classification of county of residence^†^^,^^§^^,^^¶^ — United States, December 14, 2020–April 10, 2021 * First dose of COVID-19 vaccine is defined either as the first of 2 doses for the Pfizer-BioNTech or Moderna vaccines, or a single dose for the Janssen (Johnson & Johnson) vaccine. ^†^ Excludes doses with state of residence reported as Hawaii, a territory, an island, or a county of residence in California with population <20,000. Completeness of county data varied by jurisdiction. Three states (Georgia, South Dakota, and West Virginia) had <80% completeness for county of residence data. **^§^** First doses of COVID-19 vaccine were matched by county of residence to one of six urban-rural categories according to the 2013 National Center for Health Statistics urban-rural classification scheme (https://www.cdc.gov/nchs/data/series/sr_02/sr02_166.pdf). To further classify counties into two categories (urban versus rural), four of these six categories were combined into urban areas (large central metropolitan, large fringe metropolitan, medium metropolitan, and small metropolitan) and two were combined into rural areas (micropolitan and noncore). ^¶^ Large fringe metropolitan refers to suburban areas.

## Discussion

Among most U.S. jurisdictions analyzed, COVID-19 vaccination coverage was lower overall, among all age groups, and among men and women in rural compared with urban counties. Coverage among adults aged ≥65 years was higher than among younger adults in both rural and urban areas, likely because of vaccine eligibility criteria that prioritized older adults earlier in the implementation of the vaccination program before vaccination was expanded to other age groups. Notably, vaccination coverage among women in both urban and rural areas was higher than that among men, possibly because of the increased likelihood of women seeking and using preventive care services (*6*), or women working in sectors that were prioritized for early vaccination, such as health care and education.^¶¶^ Because residents of rural communities are at increased risk for severe COVID-19–associated illness and death (*2*,*3*), vaccination disparities between urban and rural areas might hinder efforts to reduce morbidity and mortality from COVID-19 nationally.

Travel outside county of residence was used as a marker of potential vaccine access difficulties that might be exacerbated in rural areas with sparse vaccination sites. Analysis using the six-level urban-rural classification identified that a higher percentage of persons in the most rural counties traveled to nonadjacent counties for vaccination compared with those in the most urban counties, which might be related to challenges with vaccine access and the dearth of pharmacies in some rural areas (*7*). In addition, more persons in suburban (i.e., large fringe metropolitan) areas traveled outside their county of residence for vaccination; the reasons for this are unclear.

Although vaccination coverage was higher in urban counties compared with that in rural counties in most jurisdictions, five jurisdictions had similar vaccination rates between urban and rural counties and in another five, the rate in rural counties surpassed that of urban counties. Jurisdictional characteristics reported in news media that might have contributed to increased vaccination coverage in rural areas included implementing tailored approaches based on local needs, partnering with local community-based organizations and faith leaders, and engaging with underserved populations directly and through partners.***^,†††^ Local jurisdictions are collaborating with CDC to improve access to COVID-19 vaccines in rural areas by identifying and addressing barriers to vaccination. CDC is also using multiple channels to distribute vaccines, such as federal partners (e.g., the Indian Health Service and the Health Resources and Services Administration) and the Federal Retail Pharmacy program.^§§§^

Vaccine hesitancy in rural areas is a major barrier that public health practitioners, health care providers, and local partners need to address to achieve vaccination equity. In March 2021, a poll by the Kaiser Family Foundation found that vaccine hesitancy was highest in rural communities, with 21% of rural residents stating that they would “definitely not” get a vaccine compared with 10% of urban residents. Among the rural respondents, 45% of younger adults (aged 18–64 years) stated that they would “definitely not” get a vaccine compared with 8% of older adults (aged 60–69 years) (*8*). Rural residents who reported that they would “definitely not” get a vaccine were more likely to report not having a college degree and earning <$40,000 per year (*8*). Notably, 86% of rural residents report they trust their own health care providers for information on COVID-19 vaccines, which highlights the importance of public health practitioners working with established outpatient health care systems in rural areas (*9*). Through its Vaccinate with Confidence initiative, CDC continues to support rural jurisdictions and local partners in their efforts to improve access to, and bolster trust and confidence in, COVID-19 vaccines.^¶¶¶^

The findings in this report are subject to at least five limitations. First, vaccination coverage is not representative of the entire United States, because county of residence was missing for 9.2% of persons.**** Second, each jurisdiction prioritized population subgroups for vaccination differently, which might have also contributed to vaccination coverage differences between urban and rural populations. Third, COVID-19 vaccine supply changed substantially during the observed time period, and persons may have been willing to travel farther for vaccination at the beginning of this time period when vaccine supplies were low, compared with later time periods. Fourth, race and ethnicity were unknown for approximately 40% of persons with available county information; therefore, vaccination coverage could not be calculated on the basis of race and ethnicity. Improved data completeness is critical to measure and address racial and ethnic disparities in vaccination coverage. Finally, the NCHS urban-rural classification was developed in 2013, and counties that were classified as rural in 2013 might not be classified as rural during 2020–2021.

Disparities in COVID-19 vaccination between urban and rural communities can hinder progress toward ending the pandemic. Public health practitioners should continue collaborating with health care providers, pharmacies, community-based organizations, faith leaders, and local employers^††††^ to address vaccine hesitancy and ensure equitable vaccine access and distribution, particularly in rural areas (*10*). These focused, multipartner efforts can help increase nationwide vaccination coverage and reduce morbidity and mortality from COVID-19.

**Summary**

**What is already known about this topic?**

Residents of rural communities are at increased risk for severe COVID-19–associated morbidity and mortality. In September 2020, COVID-19 incidence (cases per 100,000 population) in rural counties surpassed that in urban counties.

**What is added by this report?**

COVID-19 vaccination coverage was lower in rural counties (38.9%) than in urban counties (45.7%); disparities persisted among age groups and by sex.

**What are the implications for public health practice?**

Disparities in COVID-19 vaccination access and coverage between urban and rural communities can hinder progress toward ending the pandemic. Public health practitioners should collaborate with health care providers, pharmacies, employers, faith leaders, and other community partners to identify and address barriers to COVID-19 vaccination in rural areas.

## Footnotes

*
https://www.census.gov/library/stories/2017/08/rural-america.html

† IISs are confidential, computerized, population-based systems that collect and consolidate vaccination data from providers in 64 jurisdictions nationwide and can be used to track administered vaccines and measure vaccination coverage. The 64 IIS jurisdictions comprise the 50 U.S. states, five U.S. territories (American Samoa, Guam, Northern Mariana Islands, Puerto Rico, and U.S. Virgin Islands), three freely associated states (Marshall Islands, Micronesia, and Palau), and six local jurisdictions (Chicago, Illinois; Houston, Texas; San Antonio, Texas; Philadelphia, Pennsylvania; New York, New York; and Washington, DC).

§
https://www.cdc.gov/vaccines/covid-19/reporting/vams/program-information.html

¶
https://www.cdc.gov/vaccines/covid-19/reporting/overview/IT-systems.html

** First dose of COVID-19 vaccine is defined either as the first of 2 doses for the Pfizer-BioNTech or Moderna vaccines, or a single dose for the Janssen (Johnson and Johnson) vaccine.

†† Providers are required to document vaccination in their medical records within 24 hours of administration and submit this documentation to their jurisdiction’s immunization information systems within 72 hours of administration. 5 days of observation were included to account for any delays in reporting and transmission of records to CDC.

§§ For statistical analysis, the number of doses allowed per county was capped at population size minus one for a maximum vaccination coverage of 100%.

¶¶
https://khn.org/news/article/gender-vaccine-gap-more-women-than-men-vaccinated-against-covid/

***
https://www.cnn.com/2021/03/04/health/gila-county-arizona-vaccine-trnd/index.html

§§§
https://www.cdc.gov/vaccines/covid-19/retail-pharmacy-program/index.html

¶¶¶
https://www.cdc.gov/vaccines/covid-19/vaccinate-with-confidence/strategy.html

**** Hawaii and eight California counties were excluded from analysis. More than 20% of persons receiving the first dose of a COVID-19 vaccine who live in Georgia, South Dakota, and West Virginia did not have data available for county of residence.

††††
https://www.cdc.gov/coronavirus/2019-ncov/vaccines/recommendations/essentialworker/workplace-vaccination-program.html
